# Investigating the influence of selenium and epibrassinolide on antioxidant activity, proline accumulation, and protein expression profiles in wheat plants experiencing heat and drought stress

**DOI:** 10.3389/fpls.2024.1441483

**Published:** 2024-10-22

**Authors:** Tanveer Alam Khan, Aqeel Ahmad, Taiba Saeed, Mohammad Yusuf, Mohammad Faisal, Abdulrahman Alatar Alatar

**Affiliations:** ^1^ Department of Biology, College of Science, United Arab Emirates University, Abu Dhabi, United Arab Emirates; ^2^ Key Laboratory of Land Surface Pattern and Simulation, Institute of Geographic Sciences and Natural Resources Research, Chinese Academy of Sciences (CAS), Beijing, China; ^3^ Department of Biosciences, Integral University, Lucknow, Uttar Pradesh, India; ^4^ Department of Botany and Microbiology, College of Science, King Saud University, Riyadh, Saudi Arabia

**Keywords:** abiotic stress, steroidal hormone, photosynthesis, osmoprotectant, wheat

## Abstract

In the current investigation, the combination of selenium (Se) and epibrassinolide (EBL) exhibited a promising alleviative response against the concurrent stress of heat and drought in wheat plants. The compromised growth and photosynthetic performance of wheat plants under the combined stress of heat and drought were substantially improved with the treatment involving Se and EBL. This improvement was facilitated through the expression of Q9FIE3 and O04939 proteins, along with enhanced antioxidant activities. The heightened levels of antioxidant enzymes and the accumulation of osmoprotectant proline helped mitigate the overaccumulation of reactive oxygen species (ROS), including electrolyte leakage, H_2_O_2_ accumulation, and lipid peroxidation, thus conferring tolerance against the combined stress of heat and drought. Studies have demonstrated that Se and EBL can assist wheat plants in recuperating from the adverse effects of heat and drought. As such, they are essential components of sustainable farming methods that aim to increase crop productivity.

## 1 Introduction

Plants frequently encounter many stressors at the same time, resulting in significant yield losses. As a result, it is critical to investigate the physiological and molecular mechanisms that allow plants to protect themselves against a variety of challenges. According to recent studies, plant responses to combined stresses differ from those to separate stressors and vary depending on the plant species (Zhang and Sonnewald, 2017). Moreover, plants exhibit shared reactions to stimuli and various combinations of stress factors (Zhang and Sonnewald, 2017). To delve into the distinct and widespread mechanisms governing how plants react to individual and concurrent abiotic stressors, we explored the effects of heat stress and drought, both independently and in combination, on physiological and molecular processes.

Drought and heat are two major abiotic stresses constraining wheat productivity worldwide, causing yield losses of up to 86% and 69%, respectively. Both stresses individually and in combination have a variety of negative effects since they share a lot of physiological characteristics (Prasad et al., 2011). It has recently been demonstrated that Se plays an important function in controlling plant development in both normal and severe environments. Because of its positive role in reducing the negative effects of stressors, plants require Se for development and maintenance (Yusuf et al., 2016). Selenite remains bound to the soil and is readily taken up by plants, unlike selenate, which quickly dissolves from the soil, making it unavailable for plant uptake (Fio et al., 1991). Conversely, selenium is a vital trace element necessary for plant survival, protecting detrimental environmental conditions (Feng and Wei, 2012). Selenium has been found to confer resistance to various environmental stresses such as heavy metals, drought, salinity, high temperatures, as well as bacterial and fungal infections (Schiavon et al., 2017; Muhammad et al., 2024). At low concentrations and suitable doses, it serves as an antioxidant; nevertheless, at excessive concentrations, it acts as a prooxidant (Feng and Wei, 2012; Mroczek-Zdyrska and Wójcik, 2012; Cittrarasu et al., 2021; Ikram et al., 2021). Furthermore, the plastid membrane and chloroplast need to maintain their shape and fluidity (Diao et al., 2014). Additionally, Se can promote photosynthesis (Gupta and Gupta, 2017), postpone senescence (Xue et al., 2001), and boost plant production at low concentrations (Germ et al., 2005). Other crops treated with selenium, such as *B. juncea* (Yusuf et al., 2016), *Lolium perenne* (Hartikainen et al., 2000), *S. tuberosum* (Turakainen et al., 2004), and *Lens culinaris* (Lyons et al., 2009), exhibited similar outcomes (Ekanayake et al., 2015). Brassinosteroids (BRs) represent a novel group of polyhydroxy steroidal hormones identified in plants, serving as intrinsic signals governing plant growth and expansion (Vidya Vardhini, 2017). BRs influence various developmental and physiological processes including seed germination, flowering, hypocotyl elongation, and more (Kim and Wang, 2010; Fariduddin et al., 2011; Mir et al., 2015; Dong et al., 2017; Hussain et al., 2019). Additionally, BRs exhibit the capacity to alleviate abiotic stresses such as chilling stress (Fariduddin et al., 2011), metal stress (Mir et al., 2015), elevated temperature (Hussain et al., 2019), and drought stress (Yusuf et al., 2024). Furthermore, 24-epibrassinolide (EBL), an analogue of BR, provides protective effects against environmental factors like heat stress, drought, low temperature, heavy metals, and salinity (Ali et al., 2007; Farooq et al., 2009; Khan et al., 2015; Khan et al., 2017; Khan et al., 2019; Hussain et al., 2019; Yusuf et al., 2024).

The objective of this study was to uncover the combined effect of heat and drought stress in wheat plants on growth and photosynthetic performance, antioxidant enzymes and differential expression pattern stress-responsive proteins. Additionally, dissects out stress tolerance mechanisms associated with selenium and/or brassinosteroids under combined stress of heat and drought and unlocks their contribution to the developing strategy for improving crop production under multiple stresses at a time.

## 2 Materials and methods

### 2.1 Biological material

High-quality and uniform-sized seeds of *Triticum aestivum* (wheat) cv. PBW-373 underwent surface sterilization using a half-strength standard treatment solution (composed of 15% commercial bleach and 0.01% Triton X-100) for a duration of 15 minutes. Subsequently, they were thoroughly rinsed with deionized water multiple times to remove any remaining traces of the sterilizing agents that may have adhered to the seed surface.

### 2.2 Hormone and selenium preparation

24-Epibrassinolide (EBL) was procured from Sigma–Aldrich USA. A stock solution with a concentration of 10^-4^ M was prepared and subsequently diluted to achieve the desired experimental concentrations of 10^-8^ M, as per the methodology outlined by Khan et al. (2015). Additionally, Tween-20 was employed as a surfactant by mixing with EBL before the application. Sodium selenate (Na_2_SeO_4_) served as the selenium source, with a required concentration of 10 μM prepared by diluting a stock solution (1.0 mM) of Se, a concentration chosen based on findings from our previous study (Naz et al., 2015).

### 2.3 Experimental design with treatment pattern

The surface sterilized seeds were sown in forty plastic pots, all equal size (10 inches in diameter) and volume filled with commercial potting mix and farmyard manure. These pots were arranged in a greenhouse under environmentally controlled conditions. These pots were then divided into eight sets, each containing five pots, serving as replicates for each treatment. In set I: Plants received no stress, EBL, or Se treatment, but foliage was sprayed with deionized water (served as control) at 13, 14, and 15 days of growth. Set II: Plants were exposed to 10 μM of Se through the soil at 10, 11, and 12 days of growth, and foliage was sprayed with 10^-8^ M of EBL at 13, 14, and 15 days of growth. Set III: Plants experienced a high day/night temperature of 40/35°C with a 12-hour photoperiod and 60–75% relative humidity at 20, 21, and 22 days of growth in a plant growth chamber. Set IV: Drought stress was induced by exposing plants to 15% PEG through the soil at 24, 25, and 26 days of growth. Set V – a combination of sets III and IV. Set VI – a combination of sets II and III. Set VII – a combination of sets II and IV. Set VIII – a combination of sets II, III, and IV. At the 45-day growth stage, plants from all sets were harvested to evaluate various growth parameters, leaf gas exchange traits, biochemical parameters, and protein expression. Each assay was repeated five times, with 15 plants utilized per treatment (three plants per pot).

### 2.4 Plant growth analysis

The plants were carefully removed from the pots, along with the potting mix, and subsequently rinsed with gentle running tap water to remove soil from the roots. The lengths of both the roots and shoots were then measured using a meter scale. Afterward, the plants were dried using tissue towels and placed in an oven set at 70°C for 72 hours. Following the drying process, the plant samples were allowed to cool to room temperature before their dry mass was recorded.

Leaf area was determined using a leaf area meter (AM 350, ADC Bioscientific, UK).

### 2.5 Leaf relative water content

Relative water content of leaf was determined by the method described by Fariduddin et al. (2014) by using the formula:


L RWC=(fresh mass–dry mass)/(turgidmass–dry mass)×100

### 2.6 Total chlorophyll content

Total chlorophyll content was assessed following the procedure described by Arnon (1949).

### 2.7 Determination of photosynthetic traits

To evaluate different photosynthetic characteristics including the net photosynthetic rate (*P_N_*) and stomatal conductance (*gs*), fully expanded leaves were chosen between 11:00 and 12:00 h. An infrared gas analyzer (IRGA) portable photosynthetic system (LI-COR 6400, LI-COR, Lincoln, NE, USA) was utilized for measurements. The environmental conditions such as air temperature, relative humidity, CO_2_ concentration, and photosynthetic photon flux density (PPFD) were maintained at 25°C, 85%, 600 µmol mol^-1^, and 800 µmol mol^-2^ s^-1^, respectively.

### 2.8 Determination of rubisco and carbonic anhydrase activity

Rubisco activity was assessed spectrophotometrically at 340 nm, observing the oxidation of NADH at 30°C, following the methodology outlined in our prior research (Yusuf et al., 2021). The assessment of carbonic anhydrase activity was conducted following the procedures detailed in Naz et al. (2015).

### 2.9 Assessment of stress biomarker

Lipid peroxidation, electrolyte leakage, and H_2_O_2_ content in all plant samples were quantified following the methodology outlined by Yusuf et al. (2021).

### 2.10 Assessment of protein profile and content, antioxidant enzymes, and proline accumulation

Assessment of protein content, and profile determination of antioxidant enzymes, and proline accumulation were performed according to the method mentioned in our previous study (Khan et al., 2019; Yusuf et al., 2021).

### 2.11 Statistical analysis

The data collected from the experiment underwent statistical analysis through analysis of variance (ANOVA) using IBM SPSS Statistics for Windows, Version 19.0 (IBM Corp., Armonk, NY). Results were expressed as treatment mean ± SE, and treatment means were compared utilizing the least significant difference (LSD) test at a significance level of *p ≤ 0.05*.

## 3 Experimental results

### 3.1 Growth and biomass yield

**Figure 1:**
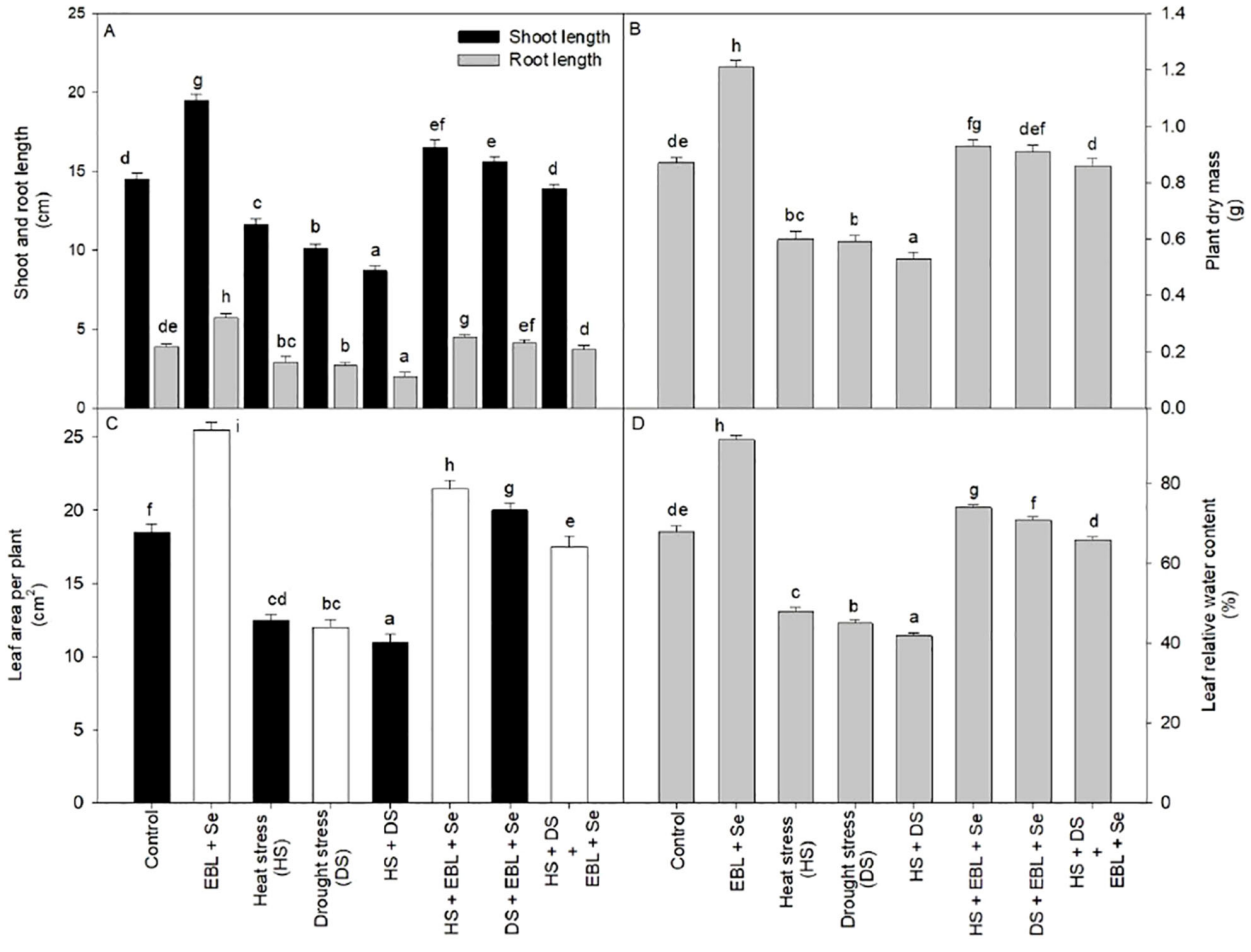
Effect of 24-epibrassinolide (EBL) in combination with selenium (Se) induced changes in **(A)** shoot and root length, **(B)** dry mass of plant, **(C)** leaf area per plant, and **(D)** leaf relative water content under combined stress of heat and drought at 45 days after sowing. All the data are the mean of five replicates (n=5) and vertical bars shows standard errors (± SE). Different letters indicate a significant difference between control and treatment by LSD test p ≤ 0.05.

Combined stress of heat and drought as well as EBL and Se showed significant effect on wheat plant height and dry biomass. However, exposure to heat stress at temperatures of 45/35°C resulted in a reduction of shoot length by 20% and root length by 25.6% compared to their respective control conditions. Moreover, when both EBL and Se were administered concurrently, there was a notable increase in shoot and root length, regardless of the stress treatments. Furthermore, the combined application of EBL and Se led to a significant enhancement in shoot length (34.4%) and root length (46.1%) compared to control plants. This combination effectively alleviated the impact of combined heat and drought stress, resulting in greater increases in plant length and dry mass compared to plants subjected to heat stress (**Figure 1**).

Similar pattern of result observed in terms of leaf area of plants.

### 3.2 Leaf relative water content

In the presence of heat, drought, or both stresses, plants demonstrated diminished relative water content in comparison to the control group. The most pronounced decline in leaf water content occurred in plants exposed to both heat and drought simultaneously, with a reduction of 38.2% compared to untreated control plants (**Figure 1D**). Conversely, the simultaneous application of EBL and Se successfully countered the loss of leaf relative water content, elevating it by 33.8% compared to the control plants.

### 3.3 Total chlorophyll content

**Figure 2:**
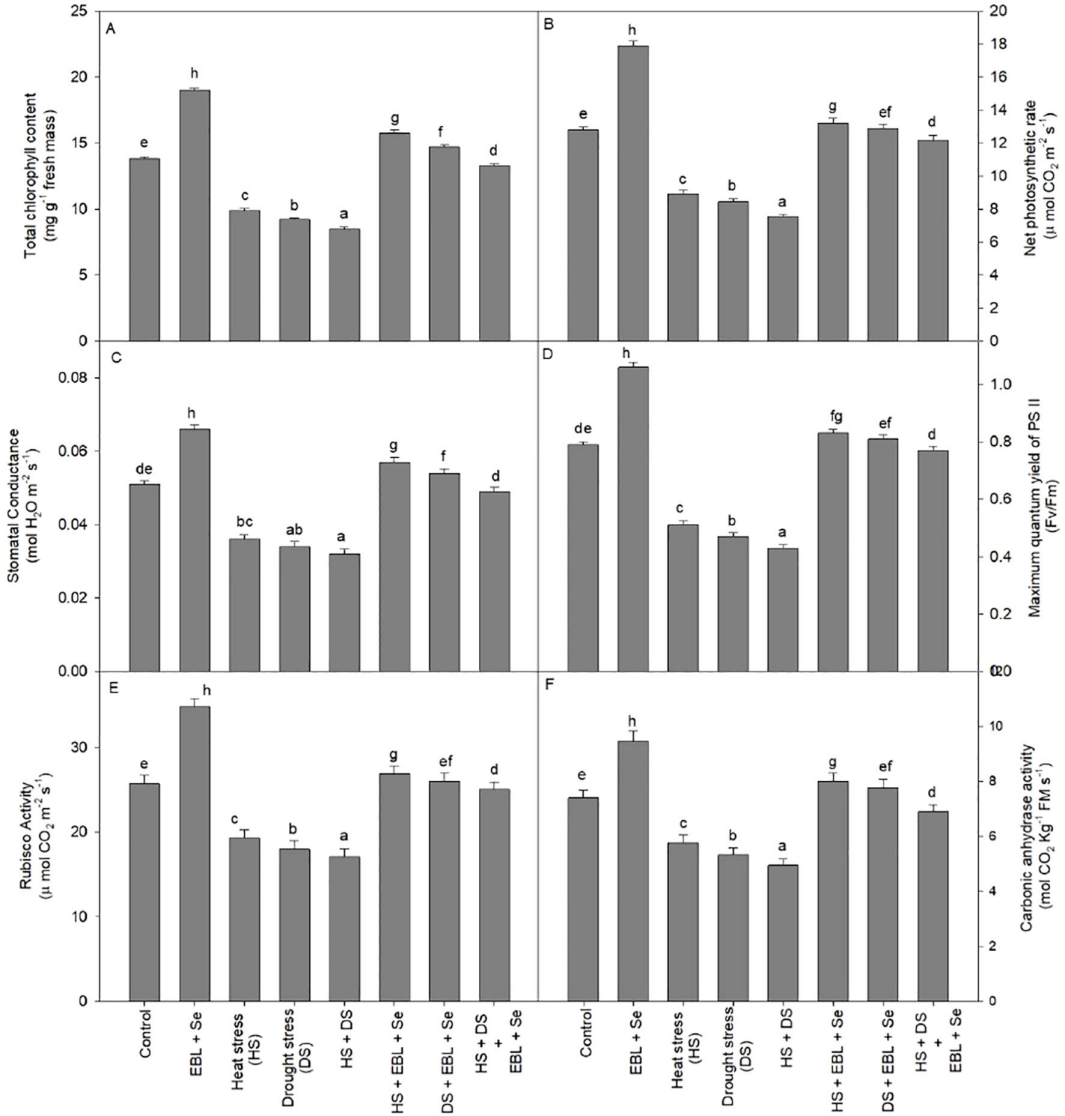
Effect of 24-epibrassinolide (EBL) in combination with selenium (Se) induced changes in **(A)** total chlorophyll content, **(B)** net photo synthetic rate, **(C)** stomatal conductance, **(D)** maximum quantum yield of PSII, **(E)** Rubisco activity, and **(F)** carbonic anhydrase activity under combined stress of heat and drought at 45 days after sowing. All the data are the mean of five replicates (n=5) and vertical bars shows standard errors (± SE). Different letters indicate a significant difference between control and treatment by LSD test p≤0.05.

When heat and dryness were combined, the plants’ chlorophyll content dropped by 38.4% when compared to the control group. On the other hand, compared to control plants, the combined application of EBL and Se dramatically raised the chlorophyll content by 37.6% (**Figure 2A**). Furthermore, the co-administration of EBL and Se significantly mitigated the reduction in chlorophyll levels caused by the combined effects of drought and heat stress.

### 3.4 Performance of photosynthetic traits

Exposure to heat, drought, or their combination resulted in notable decreases in net photosynthetic rate and stomatal conductance compared to their respective control plants (**Figures 2B, C**). The most significant reductions in net photosynthetic rate (41.4%) and stomatal conductance (37.2%) were observed in plants experiencing both heat and drought stress simultaneously. However, the concurrent application of EBL and selenium effectively addressed the challenges posed by heat and drought, restoring both net photosynthetic rate and stomatal conductance. Additionally, under non-stress conditions, the combined use of EBL and Se augmented photosynthetic characteristics beyond those observed in control plants.

The maximum quantum yield of PSII (Fv/Fm) similarly responded, protecting PSII machinery through the supplementation of EBL + Se under stressful conditions in wheat plants.

### 3.5 Rubisco and carbonic anhydrase activity

When compared to control plants, plants under heat and/or drought stress showed noticeably lower Rubisco activity (**Figure 2E**). On the other hand, Rubisco activity was greatly increased when EBL and Se were administered in combination to plants that were stressed by heat and drought. The combination showed the largest increase, with Rubisco activity rising by 35.4% in comparison to control plants. In addition, the combination treatment of Se and EBL mitigated the negative effects of heat and drought, allowing Rubisco to improve its performance.

The CA activity significantly increased by the combination of EBL and Se treatments, as shown in **Figure 2F**, and was greater by 27.9% above the control plants. However, compared to the control plants, CA activity reduced integrity by 33.1% when heat and drought stressors were present. Furthermore, in heat and drought-stressed plants, the combination of Se and EBL enhanced the activity of CA, restoring its integrity.

### 3.6 Electrolyte leakage

**Figure 3:**
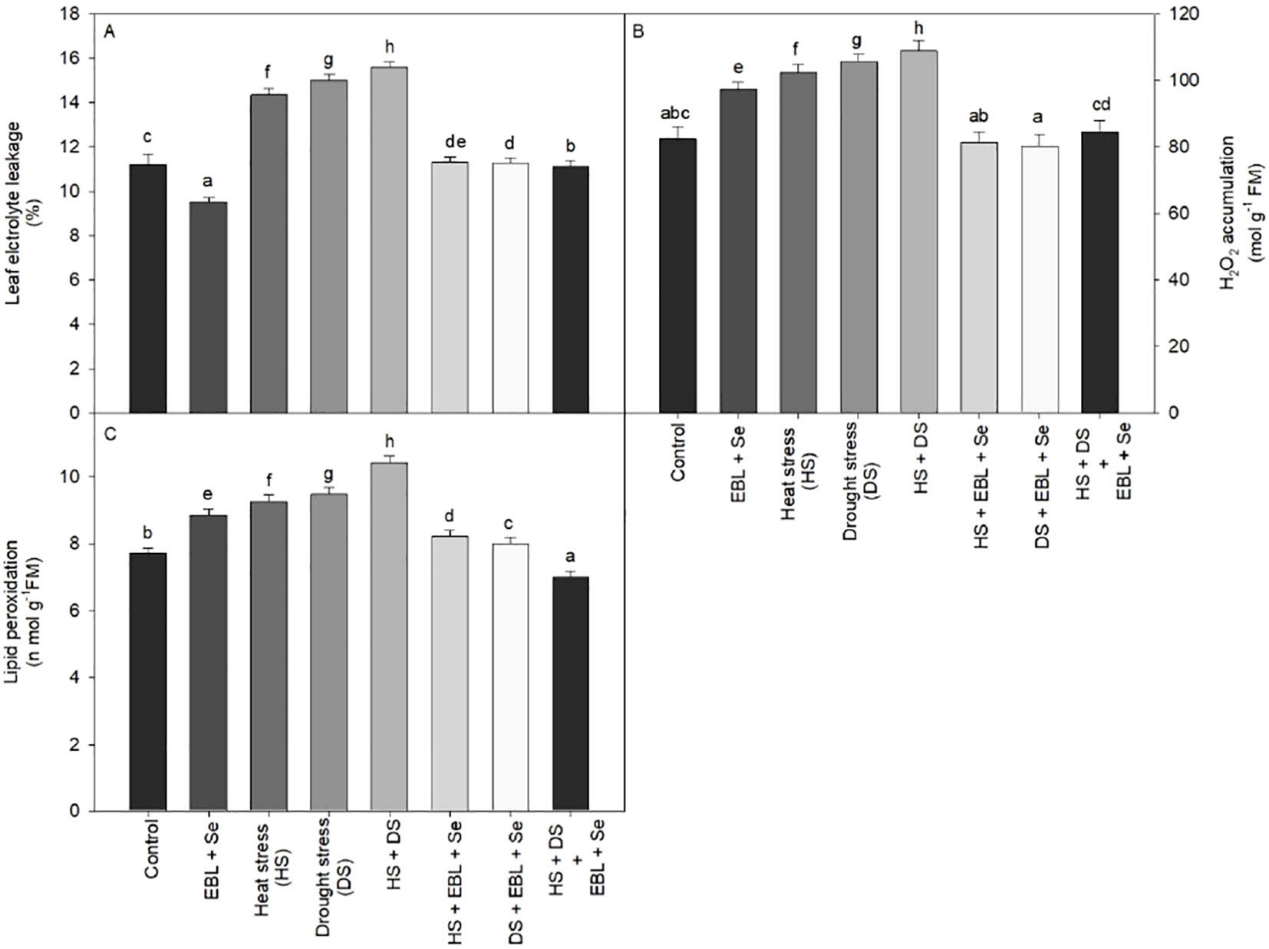
Effect of 24-epibrassinolide (EBL) in combination with selenium (Se) induced changes in **(A)** leaf electrolyte leakage, **(B)** H_2_O_2_ accumulation, and **(C)** lipid peroxidation under combined stress of heat and drought at 45 days after sowing. All the data are the mean of five replicates (n=5) and vertical bars shows standard errors (± SE). Different letters indicate a significant difference between control and treatment by LSD test p≤0.05.

Plants displayed to heat and drought stress produced more EL when compared to their respective controls, (39.2%). In non-stressed plants, however, the application of EBL and Se lowered the EL by 15.07%, respectively, when compared to the controls (**Figure 3A**). Furthermore, applying EBL and Se to plants that had been showing to heat and drought stress helped to partially reverse ion leakage.

### 3.7 Lipid peroxidation and H_2_O_2_ accumulation

As plants were exposed to heat and drought stress single or in combination, both lipid peroxidation and H_2_O_2_ buildup in leaves in contrast to their corresponding control group (**Figures 3B, C**). Heat and drought stressed plants increased LPO and H_2_O_2_ by 35.01% and. 31.99%, in compared to the control, respectively. However, LPO and H_2_O_2_ accumulation decreased in heat and drought-stressed plants when BR and Se were applied in combination as a follow-up treatment.

### 3.8 Protein content

**Figure 4:**
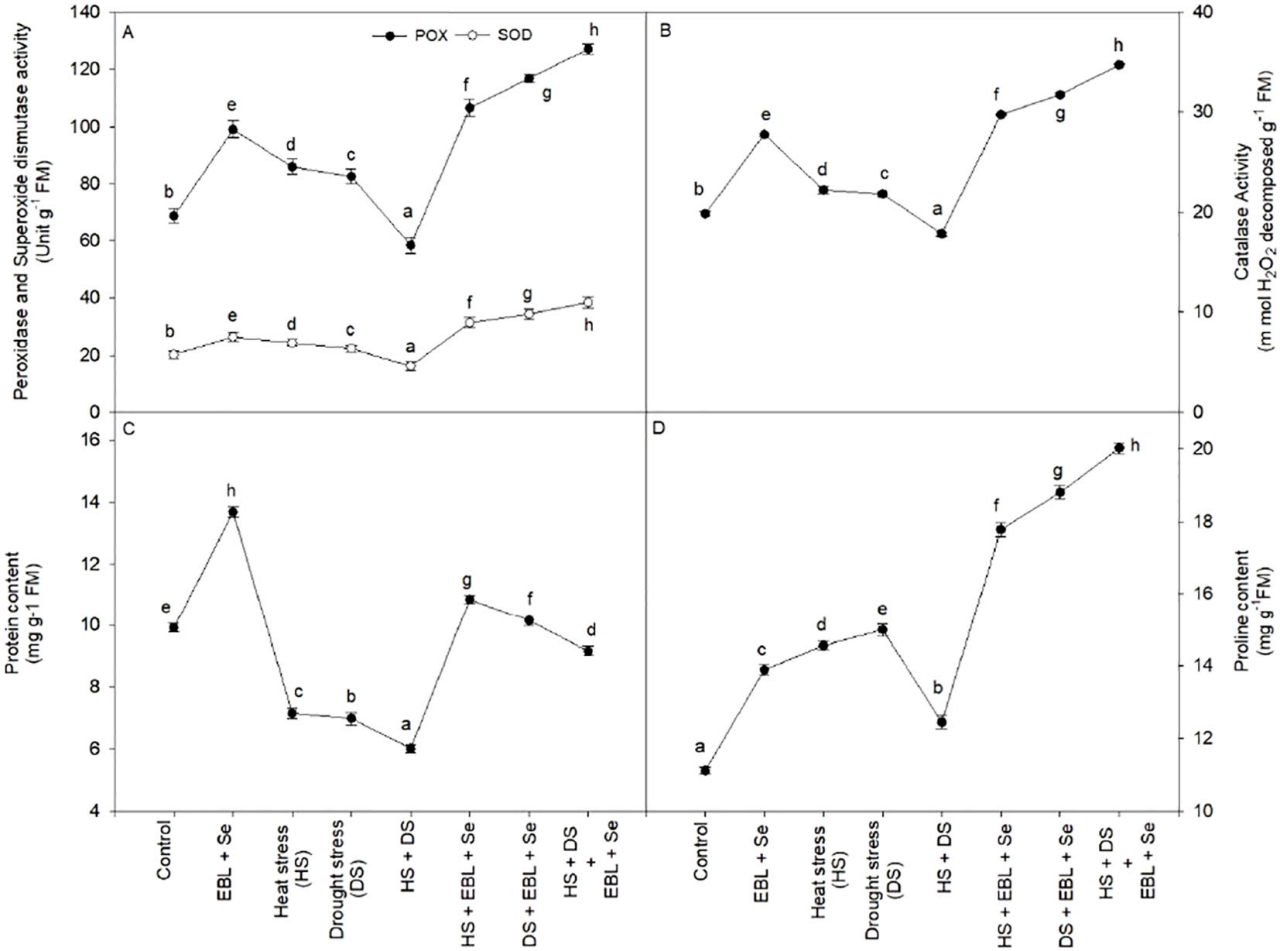
Effect of 24-epibrassinolide (EBL) in combination with selenium (Se) induced changes in **(A)** peroxidase and superoxide dismutase activity, **(B)** catalase activity, **(C)** protein content, and **(D)** proline content under combined stress of heat and drought at 45 days after sowing. All the data are the mean of five replicates (n=5) and vertical bars shows standard errors (± SE). Different letters indicate a significant difference between control and treatment by LSD test p ≤ 0.05.

When EBL + Se was given to the plants, the amount of protein in the leaves increased significantly—by 37.9% when compared to the control plants (**Figure 4C**). On the other hand, plants under stress from heat and dryness had significantly less protein than the corresponding control plants. Furthermore, the combination of Se and EBL effectively reduced the negative effects of heat and drought while increasing protein levels above those of the control plants.

### 3.9 Activities of catalase, peroxidase, and superoxide dismutase

The activity of the enzymes CAT, POX, and SOD increased dramatically in response to heat and/or drought stress as compared to the comparable control plants. However, after being subjected to a combination of heat and drought stress, wheat plants treated with EBL and Se showed the greatest increases in CAT, POX, and SOD activities (**Figures 4A, B**). When CAT, POX, and SOD readings were compared to the equivalent control plants, the increases were 75.0%, 84.9%, and 89.9%, respectively.

### 3.10 Proline content

Under the stresses of heat and drought, the leaves’ proline content rose (**Figure 4D**). In heat and drought stress conditions, proline levels increased relative to the control. Moreover, the proline content was enhanced much more after the EBL and Se treatments. The plants that were treated to heat and drought stress and then given EBL and Se treatment accumulated the maximum proline content (80.1%) as compared to their respective control plants.

### 3.11 Impact of treatments on protein profile

**Figure 5:**
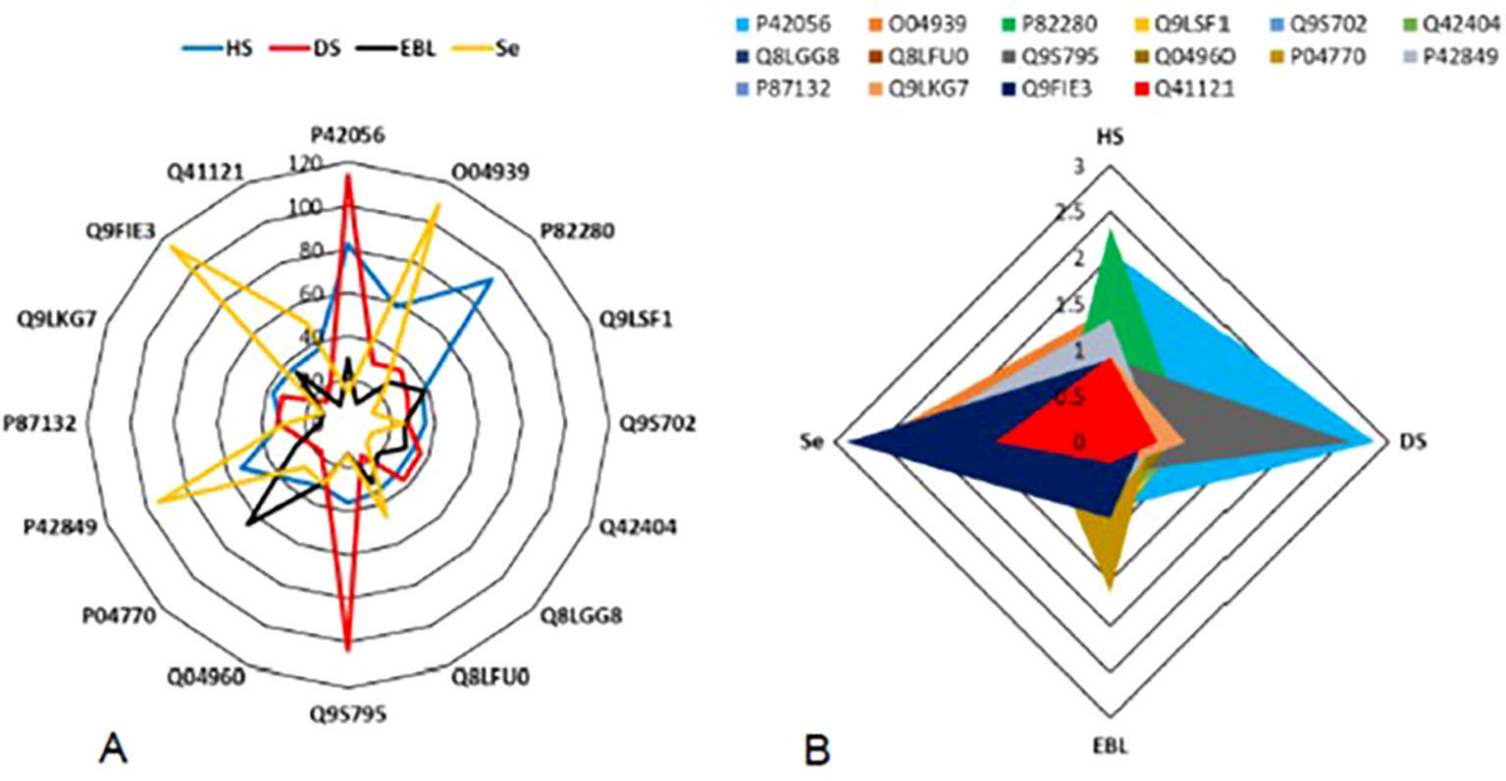
Impact of plant stressors, i.e., heat stress (HS) and drought stress (DS), and the stress alleviators i.e., epibrassinolide (EBL), and selenium (Se) on protein profile of the plant **(A)**. The correlation-based screening of differentially expressed proteins representing the affinity of the protein species with external factors **(B)**.

A varying pattern of protein expression was recorded during the comparison of protein profiles. Each treatment boosted different protein species in the plant cell. HS enhanced the expression of P82280 protein up to 80%. Whereas the protein P42056 contents were enhanced 80.7% due to HS. Protein species P42056 and Q9S795 were enhanced more than 100% (i.e., 114.5, and 107.4%, respectively) after exposing the plants with DS. Only one protein (O04939) exhibited the expression level more than 100% after having Se treatment. Selenium could elevate the expression of two protein species more than 100%, i.e., Q9FIE3 (115.7%) and O04939 (102.2%). However, an elevation of 95.4% was recorded in the protein P42849 after Se treatment. The PCA analysis screened the protein species with strongest correlation coefficients with all the external factors, i.e., HS, DS, EBL, and Se. HS had the strongest correlation coefficient of 2.3 with P82280 protein. However, P42056 exhibited the highest correlation coefficient with DS (**Figure 5**).

### 3.12 Structural analysis of protein

**Figure 6:**
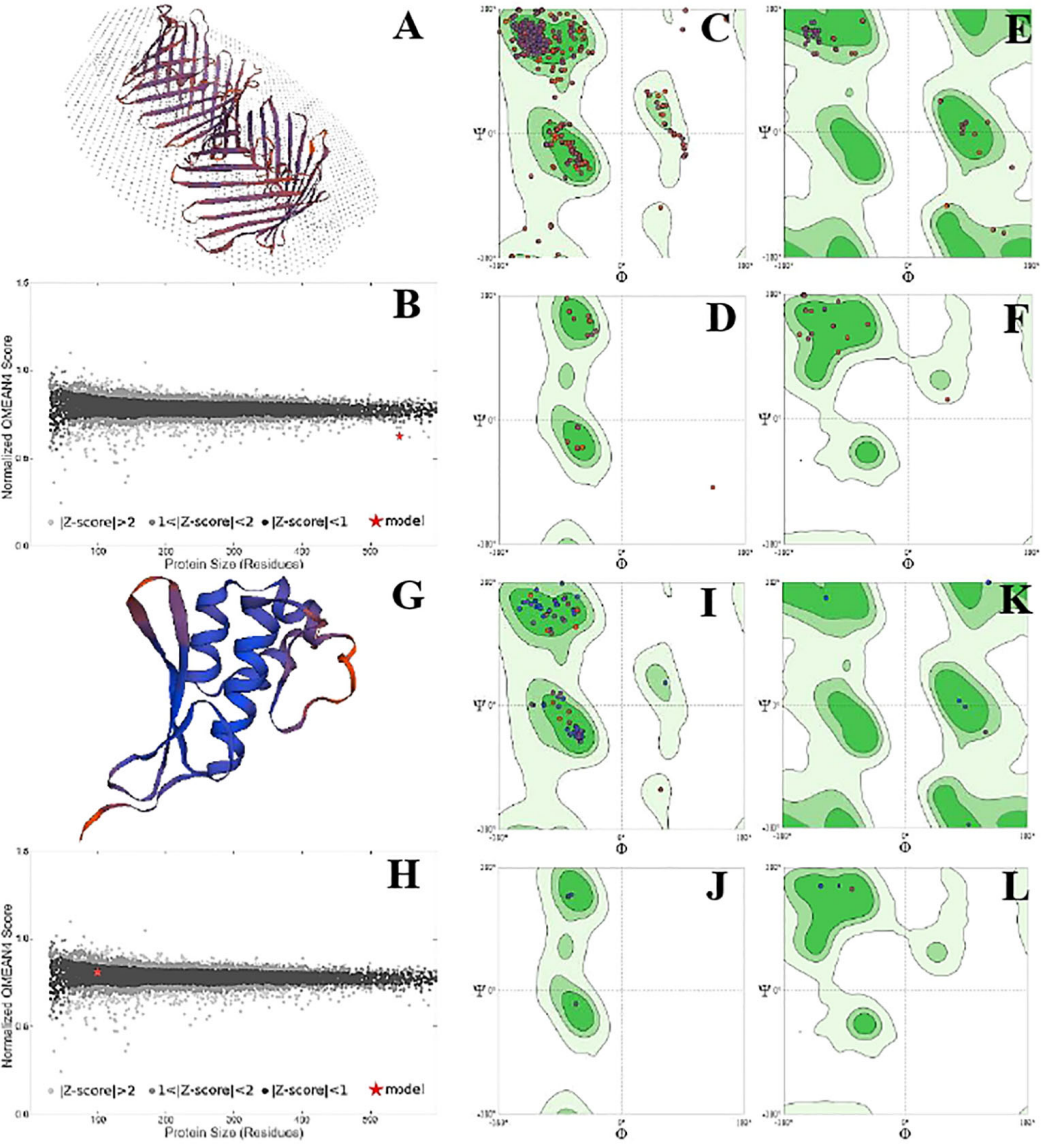
Structural analysis of protein P42056. Structural illustration of protein in ribbon model **(A)**, energy map **(B)**, the position of phi (φ) and psi (ψ) angles in the 3D structure **(C)**, The position of preproline **(D)**, the position of glycine **(E)**, and the position of proline **(F)**. Structural analysis of protein P82280. Structural illustration of protein in ribbon model **(G)**, energy map **(H)**, the position of phi (φ)and psi (ψ) angles in the 3D structure **(I)**, The position of preproline **(J)**, the position of glycine **(K)**, and the position of proline **(L)**.

**Figure 7:**
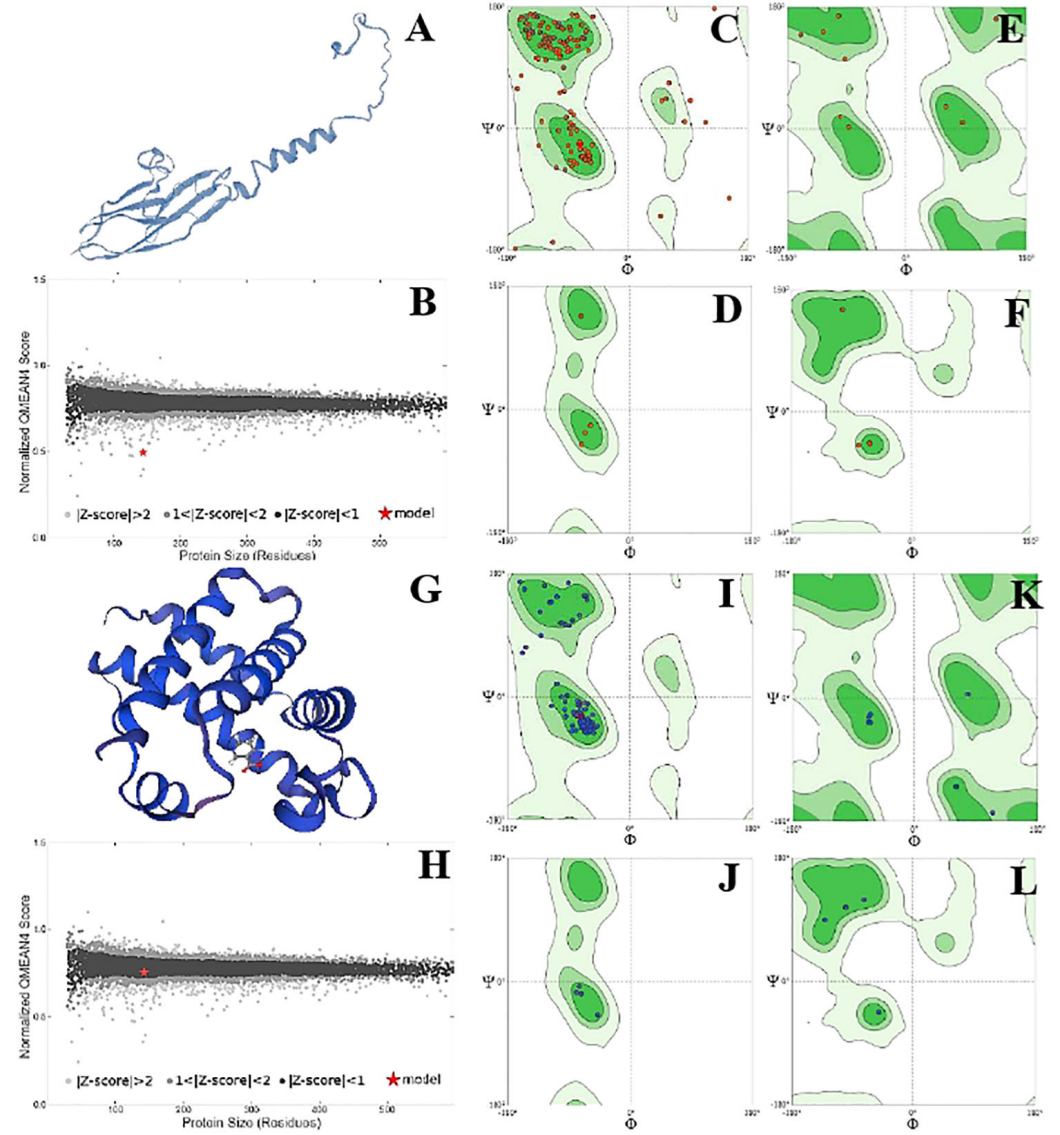
Structural analysis of protein Q9FIE3. Structural illustration of protein in ribbon model **(A)**, energy map **(B)**, the position of phi (φ)and psi (ψ) angles in the 3D structure **(C)**, The position of preproline **(D)**, the position of glycine **(E)**, and the position of proline **(F)**. Structural analysis of protein 004939. Structural illustration of protein in ribbon model **(G)**, energy map **(H)**, the position of phi (φ)and psi (ψ) angles in the 3D structure **(I)**, The position of preproline **(J)**, the position of glycine **(K)**, and the position of proline **(L)**.

The position of phi (φ) and psi (ψ) angles illustrated the detailed activity and stability of the protein species. Overall, protein species P42056 had a significantly higher number of C-N-C angles than protein species P82280. It showed the increased number of active sites on the outer surface of the globular protein. However, the structure of the P82280 protein was more stable than the P42056. Its stability was evident from its small structure, energy level, and the presence of a higher number of proline residues in the structure. Glycine residues with C-N-C bonding were also more in number in the P42056 as compared to protein species P82280. Protein P82280 exhibited no C-Ca-C proline residue (**Figure 6**). In addition to this, protein species Q9FIE3 had a linear structure with one lobed end. Its long linear structure made it an unstable structure with 0.5 QMean energy score. There was more density of phi (φ) and psi (ψ) angles in protein Q9FIE3. Furthermore, the ratio of N-C-N angles was significantly higher than C-Ca-C angles. All the glycine residues were detected in the upper two quadrates (1^st^ and 4^th^) of the Ramachandran plot. Similarly, proline and preproline residues were present in the 3^rd^ and 4^th^ quadrates. Protein species O04939 had a globular and stable structure. It’s QMean score was 0.78. The preproline contents were detected in the 3^rd^ quadrate only (**Figure 7**).

## 4 Discussion

In various abiotic conditions, lipid peroxidation, H_2_O_2_ accumulation, and electrolyte leakage serve as biochemical markers indicative of damage caused by free radicals (Verma and Dubey, 2003). Additionally, heightened production of reactive oxygen species (ROS) is a common characteristic of these metabolic processes (Foyer and Noctor, 2003; Gill and Tuteja, 2010). Our findings support this notion, showing an increase in lipid peroxidation, H_2_O_2_ accumulation, and electrolyte leakage due to heat and drought stress (**Figure 3**). Consequently, oxidative stress is believed to underlie the damage incurred by plants due to heat and drought. According to earlier research, oxidative bursts and the production of ROS are brought on by heat and drought stress. Overproduction of ROS during abiotic stress conditions upsets the balance between ROS generation and removal (del Río et al., 2006; Navrot et al., 2007). ROS in plant tissues are regulated by osmoprotectants like proline, non-enzymatic low molecular weight antioxidants like glutathione, carotenoids, and tocopherols, and antioxidant enzymes like superoxide dismutase, catalase, peroxidase, and glutathione reductase (Schützendübel and Polle, 2002; Szabados and Savouré, 2010). Despite both Se and ROS serving as important signaling molecules in plants, their effects on plant damage, growth, and stress resistance differ significantly (Apel and Hirt, 2004). Conferring to our current research, applying Se and EBL to plants boosted proline accumulation (**Figure 4D**) and antioxidant enzyme activity (**Figure 4**), regardless of whether heat and drought stress were present. Additionally, plants under stress from heat and drought displayed the highest values; these plants were then given a combination of EBL and Se (**Figure 4**).

To combat the excessive oxidative stress induced by ROS, plants have evolved a number of complicated cellular and systemic homeostatic systems. These processes include both non-enzymatic defense like free amino acids, particularly proline, and enzymatic defenses such as SOD, CAT, and POX. These levels were even higher after Se and EBL treatment. Plants treated with EBL and Se under drought stress conditions showed the highest activity of CAT, POX, and SOD (**Figure 4**). Furthermore, a great deal of research has indicated that selenium (Se) might be a quasi-essential element that plants need to provide defense against a range of abiotic stressors, including salt (Hawrylak-Nowak, 2009) and heavy metals (Mroczek-Zdyrska and Wójcik, 2012). The enhanced expression of the *det2* gene is responsible for the EBL-induced boost of antioxidant enzymes. This gene improves the antioxidant system, hence increasing Arabidopsis tolerance to oxidative stress (Cao et al., 2005). Our findings support previous research that found that exogenously administered BRs altered antioxidant enzyme activity in response to diverse abiotic stressors (Khan et al., 2019). Proline is thought to work as a signaling molecule, regulating mitochondrial processes and triggering particular gene expression, which may be necessary for plants to recover from abiotic challenges (Szabados and Savouré, 2010). In line with these findings, the current study discovered that extreme heat and drought in the presence of both EBL and Se increased proline accumulation significantly (**Figure 4D**). Furthermore, proline metabolism influences a variety of developmental and stress responses, and its accumulation contributes in providing tolerance to a wide range of environmental stressors (Szabados and Savouré, 2010). Under stress, BRs can increase proline buildup by expressing genes involved in proline biosynthesis (Ozdemir et al., 2004; Zeng et al., 2010). Additionally, it was demonstrated that applying Se to *Brassica napus* plants increased both the enzymatic and non-enzymatic antioxidant systems, hence strengthening the plants’ resistance to oxidative damage caused by metals (Hasanuzzaman et al., 2012).

Our current investigation found that growth metrics, such as leaf area, fresh and dry mass of shoot and root, and root and shoot length, were considerably reduced under heat and drought stress conditions (**Figures 1A–D**). The relationship between heat and drought stress is well-established, since lower transpiration and stomatal conductance during droughts can make heat stress worse by raising leaf temperatures (Lipiec et al., 2013). In the field, rapid declines in plant growth and productivity can result from simultaneous heat waves and droughts, particularly in tropical and subtropical locations (Niinemets, 2016; Zandalinas et al., 2017). Increased temperatures often result in changes to the structure of leaves, which are characterized by increased surface areas and thinner leaves (Luomala et al., 2005; Poorter et al., 2009). Although Wahid et al. (2007) found comparable effects on cell density across high temperature and drought stress, there is still a dearth of knowledge about how high temperature affects leaf architecture (Wahid et al., 2007). In line with earlier research, our findings showed that supplementing wheat with low dosages of Se increased its growth, leaf area, and chlorophyll content (Naz et al., 2015; Yusuf et al., 2016). Comparable results have been noted for ryegrass, lettuce, wheat, and *B. juncea*, among other plant species (Hartikainen et al., 2000; Ramos et al., 2010; Naz et al., 2015; Boldrin et al., 2016; Yusuf et al., 2016).

Furthermore, as suggested by Xie et al. (2011), the transcriptional impacts of brassinosteroids (BR) and their influence on biomass could prove instrumental in enhancing agricultural yields and resilience. BR signaling governs both cell growth and proliferation, crucial processes for biomass production. By directly modulating the expression of *CELLULOSE SYNTHASE* (*CESA*) genes in *A. thaliana*, BR promotes plant development through cell elongation, thereby augmenting cellulose content and biomass accumulation (Xie et al., 2011). Heat stress impedes cell elongation and induces cell cycle arrest by downregulating genes such as *CESA* and certain cyclins (Guerriero et al., 2014; Zhao et al., 2014). Together with *CESA*, BR also increases the production of enzymes that aid in the expansion and loosening of cell walls, such as expansins, xyloglucan endotransglucosylase, and pectin-lyase (Uozu et al., 2000; Guo et al., 2019). Considering that BR can rescue cell growth from heat stress (Hatfield and Prueger, 2015). BR can boost crop biomass and overall production in both favorable and stressful conditions by targeting *CESA* gene and its homologs. In addition, the fresh and dry weight of wheat plants were raised by the foliar application of EBL (Bajguz and Tretyn, 2003).

The use of EBL in combination with Se considerably improved SPAD chlorophyll levels, photosynthetic machinery, and photosystem II (Fv/Fm) in the current study (**Figure 2**). In our current investigation, both heat and drought stress exerted a significant adverse impact on photosynthesis-related parameters, leading to a reduction in chlorophyll levels and photosystem II productivity (**Figure 2**). The onset of senescence and changes in the activity of interconnected enzymes are believed to induce changes in cytoplasmic composition and modulate sink activity, thereby impeding the transport of photosynthates and ultimately decreasing the rate of photosynthesis (Cakmak, 2005). Earlier research has demonstrated that heat, salt, and water stress reduce the effectiveness of transpiration rates and photosynthesis, either separately or in combination (Arbona et al., 2013; Zandalinas et al., 2016). The main cause of this decrease is stress-induced stomatal closure, although non-stomatal restrictions including decreased leaf growth, senescence, and compromised photosynthetic apparatus performance can also have an impact (Saibo et al., 2009; Rahnama et al., 2010). In the latter case, decreased internal CO_2_ availability combined with suppression of essential photosynthetic enzymes and ATP synthases is frequently implicated. It has been demonstrated that heat and dryness inhibit electron transport, cause protein degradation, and liberate calcium and magnesium ions from protein-binding partners (Wahid et al., 2007; Rexroth et al., 2011; Zlatev and Lidon, 2012; Zandalinas et al., 2016).

As a result, the current investigation revealed that plants treated with Se had dramatically improved *P_N_*, and *gs*, in both stressed and non-stressed environments. The potential rationale for using Se was to enhance photosynthetic efficacy by influencing the activation of defensive mechanisms in plants at various levels. Furthermore, Se treatment has been shown to significantly increase photosynthesis by upregulating chlorophyll synthesis (Zhang et al., 2014; Naz et al., 2015; Yusuf et al., 2016) and photosynthetic characteristics (*P_N_*, and *gs*) (Zhang et al., 2014; Naz et al., 2015; Yusuf et al., 2016). Se treatment enhanced the leaf area, total chlorophyll content, photosynthesis-related characteristics, and photosystem II in *B. juncea* during stress conditions (Yusuf et al., 2016). Additionally, EBL-treated plants had improved photosynthetic parameters (*P_N_*, and *gs*) to improve CO_2_ adjustment and increased the efficiency of the light-picking complex by raising chlorophyll content. The present findings align with the research undertaken by other investigators, demonstrating that the exogenous application of BRs enhanced photosynthesis and associated characteristics, as well as the quantum yield of PSII under stressful conditions (Khan et al., 2015; Yusuf et al., 2017).

Several research explained the severe effects of environmental stressors on agricultural crops (Khan et al., 2019; Shah et al., 2020; Ahmad et al., 2021; Tariq et al., 2021). Besides considering the deleterious roles of drought and heat, it is very important to study the physiological mechanisms of the stressors. It is important to study the proteins behind the plant stressor impacts (Khan et al., 2018; Shah et al., 2020). Therefore, the current study is a precious advancement because it provides a detailed view of the proteins involved in heat and drought stress. Furthermore, the study also gives a comprehensive view of the stress alleviators. The proteins involved in stress alleviation can be used to eliminate the stress effects from the plants (Shah et al., 2020; Tariq et al., 2021). For that purpose, their expression can be enhanced in the plants. Drought and heat are the two trenchant factors severely affecting our crops and their production (Ahmad et al., 2021). There are multiple reports of complete crop failure due to severe drought hits or abrupt heat shocks. It is very important to study the molecular mechanisms of these environmental factors to cope with the changing climate. Further studies can help to develop resistant cultivars against drought and heat. Proteins are the functional unit of the cellular processes (Bashir et al., 2016). Previously several proteins have been reported to deal with external stimuli (Shafique and Ahmad, 2013; Ahmad et al., 2014; Akram et al., 2014; Ahmad et al., 2019). Similarly, a single protein may be involved in dealing the multiple stimuli. The current study first time reports two stress-related proteins and two stress alleviation-related proteins. Furthermore, the study provides a full report on their structural and functional stability, allowing researchers to pick stable proteins for stress-related research. The proteins can then be introduced into new cultivars to help them adapt to changing climate conditions. To do this, genetic engineering and other molecular approaches can add protein-encoding genes into new cultivars or plants, increasing their resilience to drought and heat stress.

## 5 Conclusion

The combined application of Se and BRs demonstrated encouraging alleviative effects in wheat plants subjected to concurrent heat and drought stress, evidenced by the upregulation of specific proteins (Q9FIE3 and O04939) and enhanced synthesis of primary antioxidant enzymes (catalase, peroxidase, and superoxide dismutase) to counteract ROS. These responses were effectively reflected in the growth performance and photosynthetic efficiency of wheat plants under the combined stress of heat and drought. This study revealed new and important insights into the potentialities of Se and BRs mediated amelioration of combined stress of heat and drought through modulating protein expression, and enhanced production of antioxidants, and osmolytes along with improved photosynthetic performance. Moreover, for the first time in this study, two stress-related proteins and two stress-relieving proteins are reported in response to EBL, Se, heat, and drought stress. Furthermore, the study provides a full report on their structure and functional stability, enabling researchers to choose stable proteins for stress-related studies. The gene responsible for the expression of these proteins could be incorporated into the new cultivars to cope with changing climate. For that purpose, genetic engineering and other molecular techniques can introduce protein-encoding genes into new cultivars or plants to enhance their resistance to drought and heat stress.

## Data Availability

The raw data supporting the conclusions of this article will be made available by the authors, without undue reservation.
